# Direct evidence of the use of multiple drugs in Bronze Age Menorca (Western Mediterranean) from human hair analysis

**DOI:** 10.1038/s41598-023-31064-2

**Published:** 2023-04-06

**Authors:** E. Guerra-Doce, C. Rihuete-Herrada, R. Micó, R. Risch, V. Lull, H. M. Niemeyer

**Affiliations:** 1 Departamento de Prehistoria, Arqueología, Antropología Social y Ciencias y Técnicas Historiográficas, Universidad de Valladolid, Plaza del Campus sn, 47011 Valladolid, Spain; 2 Departament de Prehistòria, Facultat de Lletres, Carrer de la Fortuna, Universitat Autònoma de Barcelona, 08193 Bellaterra (Cerdanyola del Vallès), Barcelona, Spain; 3 Departamento de Química, Facultad de Ciencias, Universidad de Chile, Casilla 653, Santiago, Chile

**Keywords:** Diagnostic markers, Mass spectrometry, Organic chemistry, Archaeology, Archaeology

## Abstract

Human hair dated to Late Prehistory is exceedingly rare in the Western Mediterranean. Archaeological excavations in the Bronze Age burial and cult cave of Es Càrritx, in Menorca (Balearic Islands) provided some human hair strands involved in a singular funerary rite. This finding offered the opportunity to explore the possible use of drug plants by Late Bronze Age people. Here we show the results of the chemical analyses of a sample of such hair using Ultra-High-Performance Liquid Chromatography-High Resolution Mass Spectrometry (UHPLC-HRMS). The alkaloids ephedrine, atropine and scopolamine were detected, and their concentrations estimated. These results confirm the use of different alkaloid-bearing plants by local communities of this Western Mediterranean island by the beginning of the first millennium cal BCE.

## Introduction

Human consumption of drug plants is a long-standing tradition^1–3^. By combining many different fields of study (Archaeology, Anthropology, Chemistry, Pharmacology, Ethnobotany, and Iconography, among others) it has been possible to trace back this habit to prehistoric times^4–7^ in Eurasia^8–15^, North America^16–19^ and South America^20–26^. As mind-altering substances are usually invisible in the archaeological record, their presence used to be inferred from indirect evidence, such as the typology and function of certain artefacts possibly related to their preparation or consumption (pottery vessels, stone mortars, snuffing kits, smoking pipes, and enema syringes, among others)^27–32^ and botanical remains (macro and microfossils) of drug plants^33–36^.

Also, since psychoactive agents can remain preserved for millennia, chemical analysis of archaeological residues may provide indirect evidence of the consumption of drugs in the past. Thus, opium alkaloids were detected in Late Bronze Age containers from the eastern Mediterranean^37,38^, providing chemical evidence to support the hypothesis that the shape of these juglets as inverted poppy capsules served to advertise their contents^31^. Opium and tropane alkaloids were reported by J. Juan-Tresserras in Chalcolitic, Bronze Age and Iron Age containers from Iberia^39–41^ (however, the lack of clarity in detailing methodological procedures have affected the trustworthiness of these results); different hallucinogenic compounds, mainly nicotine, tryptamines and tropane alkaloids have been chemically documented in Prehispanic artefacts from the Americas^42–61^, and psychoactive compounds of Cannabis in archaeological wooden braziers from China^62^.

Direct evidence of the intake of drugs by ancient populations derives from chemical analysis of human remains. These analyses have revealed the presence of psychoactive alkaloids in hair samples of American Prehispanic mummified individuals^63–74^, in human bones from prehistoric China^75^ and from Late Neolithic variscite mines at Gavá, near Barcelona^76^, and in skeletons from south Germany Bell Beaker culture^77,78^ (some of these results are controversial. The methodological procedures applied to the Gavá skeletons is poorly described in the publication and the findings of certain alkaloids in ancient Egyptian mummies^79–81^ have been widely criticized for the analytical techniques employed^82,83^, and consequently the consistency of the methods and the interpretation of the results have been much debated). Furthermore, Areca nut alkaloids were detected in the dental enamel of Iron Age Vietnam individuals^84^.

The recovery of human hair in a Late Bronze Age burial cave in Menorca, in the Balearic Islands, provided a unique opportunity to further probe into the medicinal and ritual realms of indigenous inhabitants of the Western Mediterranean as early as 3,000 years ago through the analysis of its alkaloid content. The results furnish direct evidence of the consumption of plant drugs and, more interestingly, they reveal the use of multiple psychoactive species.

### The archaeological context: The ritual and funerary cave of Es Càrritx, in Menorca

**Figure 1:**
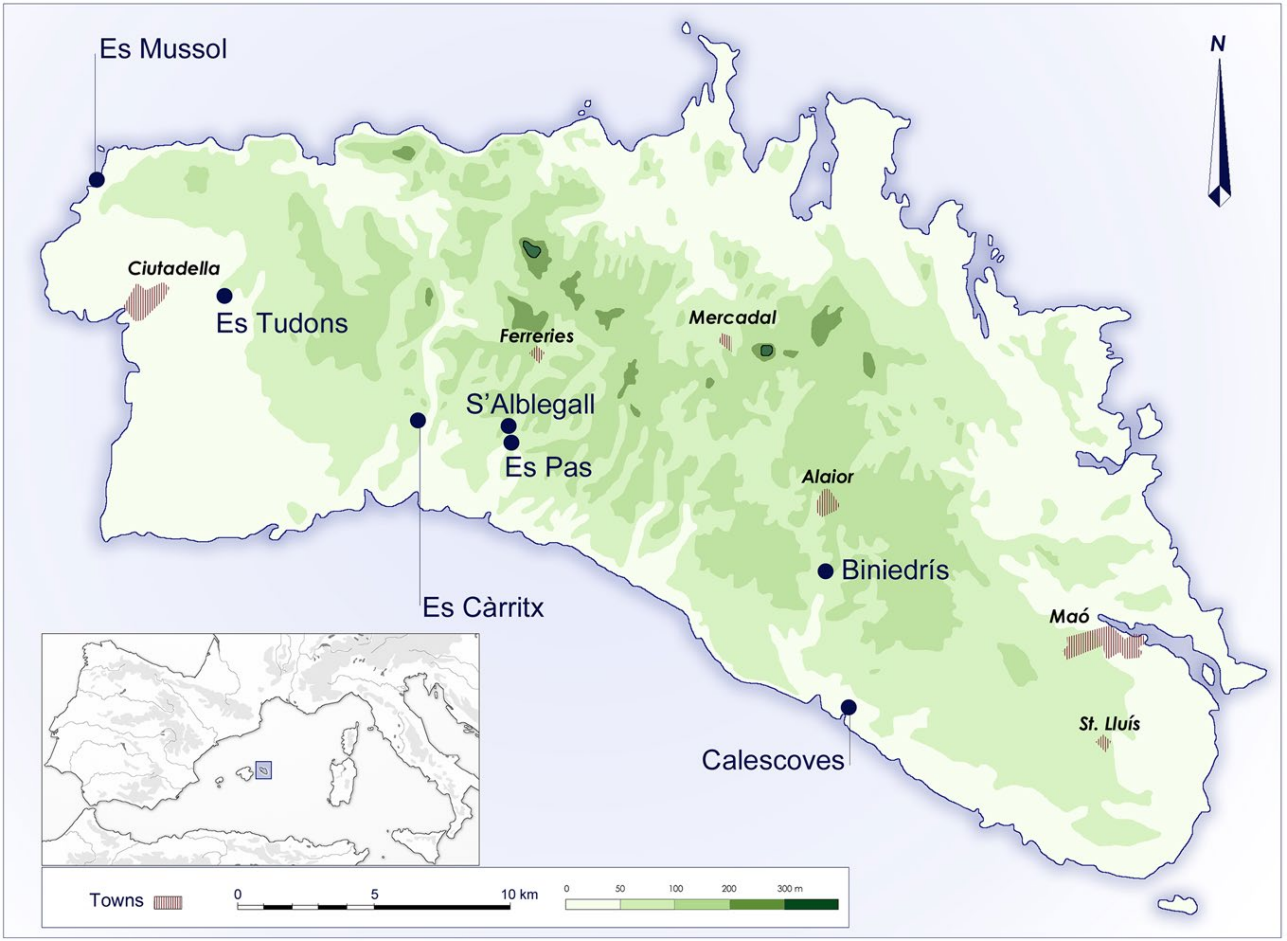
Location of the funerary sites mentioned in this study.

The early colonization of the Balearic Islands is a complex issue^85^. At least the two larger islands (Mallorca and Menorca) of this Western Mediterranean archipelago were only inhabited permanently from the second half of the third millennium BCE, during the Late Copper Age/Early Bronze Age^86,87^. By the beginning of the second millennium cal BCE, the settled islanders began the development of monumental stone structures for funerary purposes, such as dolmens, megaliths, cairns, and rock-cut tombs, and by *ca*. 1600 cal BCE they constructed *navetes* or boat-shaped habitational structures^88^. Around *ca*. 1450 cal BCE a new type of funerary structure appeared: natural caves whose entrances were closed with the same type of cyclopean walls as used for the construction of domestic *navetes*. One of these caves is the cave of Es Càrritx, in Menorca, discovered intact by the speleologists Pere Arnau and Josep Márquez in 1995 (Fig. 1).

**Figure 2:**
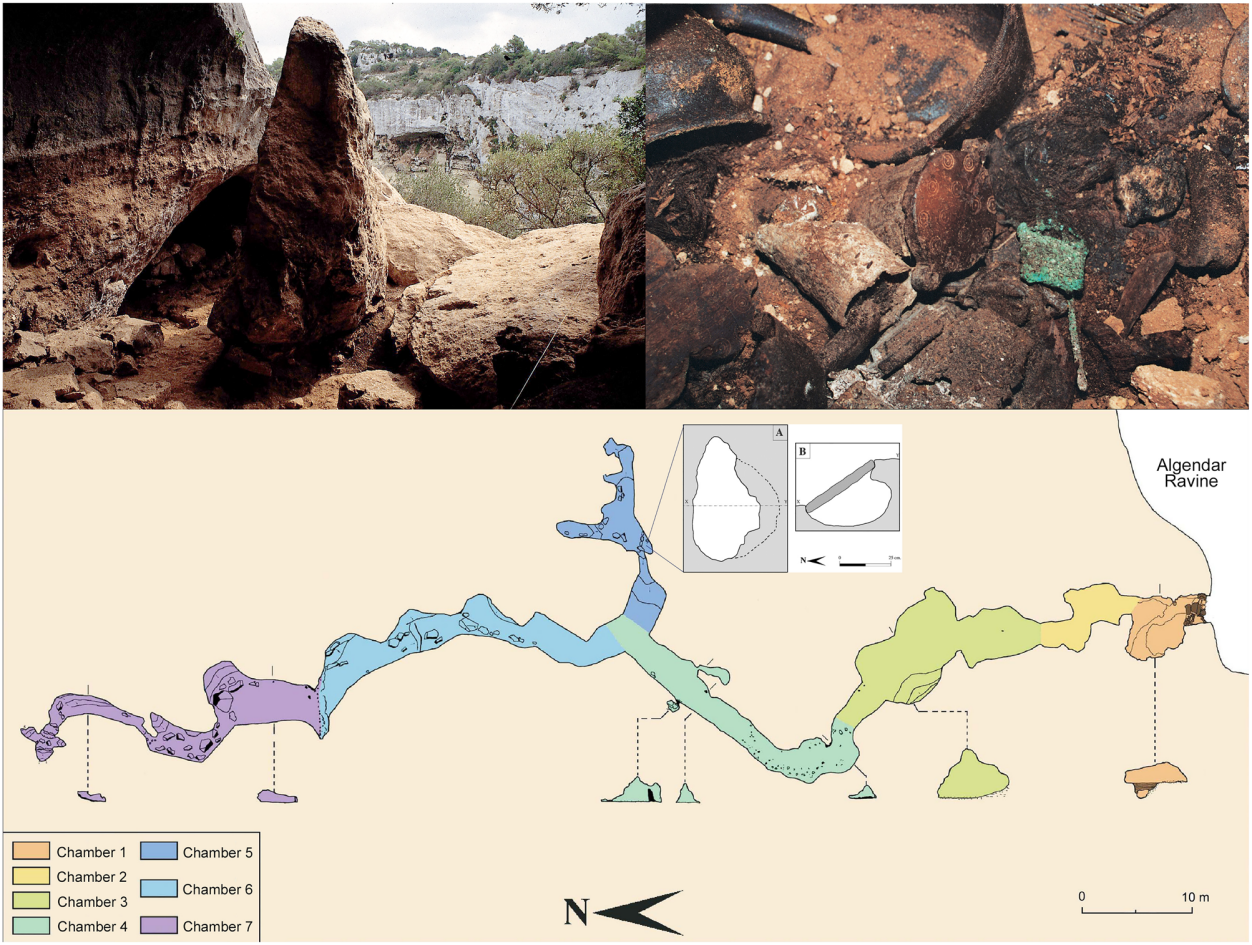
View of the entrance of Es Càrritx (upper left); the deposit of Chamber 5 with the tubes containing the human hair placed at the center (upper right, courtesy of Consell Insular de Menorca); plan of the cave and section of the deposit found in chamber 5 (P. Arnau, J. L. Florit, J. Márquez & M. Márquez).

The cave is located at the Algendar ravine (39º57′59.22″ N–3º57′54.828″ E, 55 m a.s.l.) and it is one of the most important Late Bronze Age (locally known as Naviform period) sites on the island. The gorge is ca. 90 m deep, and the cave entrance is located some 25 m from the top of the cliff. It was first occupied *ca*. 1600–1500 cal BCE when it housed ritual activity. At the onset of the Middle Naviform period (ca. 1450/1400 cal BCE), chamber 1 located at the entrance of the cave became a collective funerary space and continued serving this function for nearly 600 years until *ca.* 800 cal BCE^89^ (Fig. 2). The funerary space accommodated the bodies of over two hundred individuals of both sexes and all age groups except for fetuses—implying that no pregnant women were buried there—and babies under three months. Osteological data and palaeodemographic calculations show that closely related members of a social unit of *ca*. 14 individuals were buried in this cave generation after generation^90^.

At the cave of Es Càrritx, but also at other burial sites in Menorca (e.g., Cova des Pas, some of the hypogea at the Calescoves cemetery, Biniedrís cave, the *naveta* of Es Tudons), there is evidence that during a 300-year period before the final use of these tombs (between *ca.* 1100 and 800 cal BCE, according to radiocarbon dating results) a singular *post-mortem* treatment took place involving part of the deceased individuals’ hair^91^. After the corpses were deposited, strands of hair were intentionally dyed or anointed red in situ. Hematite-rich ochre pigments may have been used, as in the Biniedrís cave^92^, or possibly extracts of some plants traditionally exploited for red dye, such as wild madder (*Rubia peregrina*) or Balearic box (*Buxus balearica*), both present in the archaeobotanical funerary record of the cave of Es Càrritx^93,94^. Subsequently, some locks of hair were combed, cut out, and finally introduced into tubular containers made of wood or antler provided with bases and lids which sealed the hair strands inside. The lids, often decorated with carved series of perfect concentric circles, held opposing perforated lugs that served to secure the containers with the aid of strings.

Once the ritual was completed, the tubes containing hair were usually left nearby the deceased. However, at the cave of Es Càrritx a group of artefacts involved in the ceremony were removed from Chamber 1 and hidden inside a hoard in Chamber 5, a small space deep in the cave that had remained sealed since *ca*. 800 cal BCE^89^. The assemblage was made up of six complete wooden containers, four complete horn containers, four wooden spatulas, four wooden canes, one wooden stick, three wooden vessels, one wooden comb, two ceramic vessels, and some bronze items (a blade, a hairpin and part of the rod of a second pin). It appears that the objects were intentionally hidden together by depositing them at a single event inside a pit that had been excavated in the natural clay of the cavity, and then covered with a slab of compacted clay. The depositional sequence indicates that the tubes were placed at the center, and the other objects were disposed around them. The fact that containers found in Chamber 5 (n = 10) were largely fewer than the number of individuals found in Chamber 1 (MNI = 210) suggests that these rituals were performed only with selected individuals.

**Figure 3:**
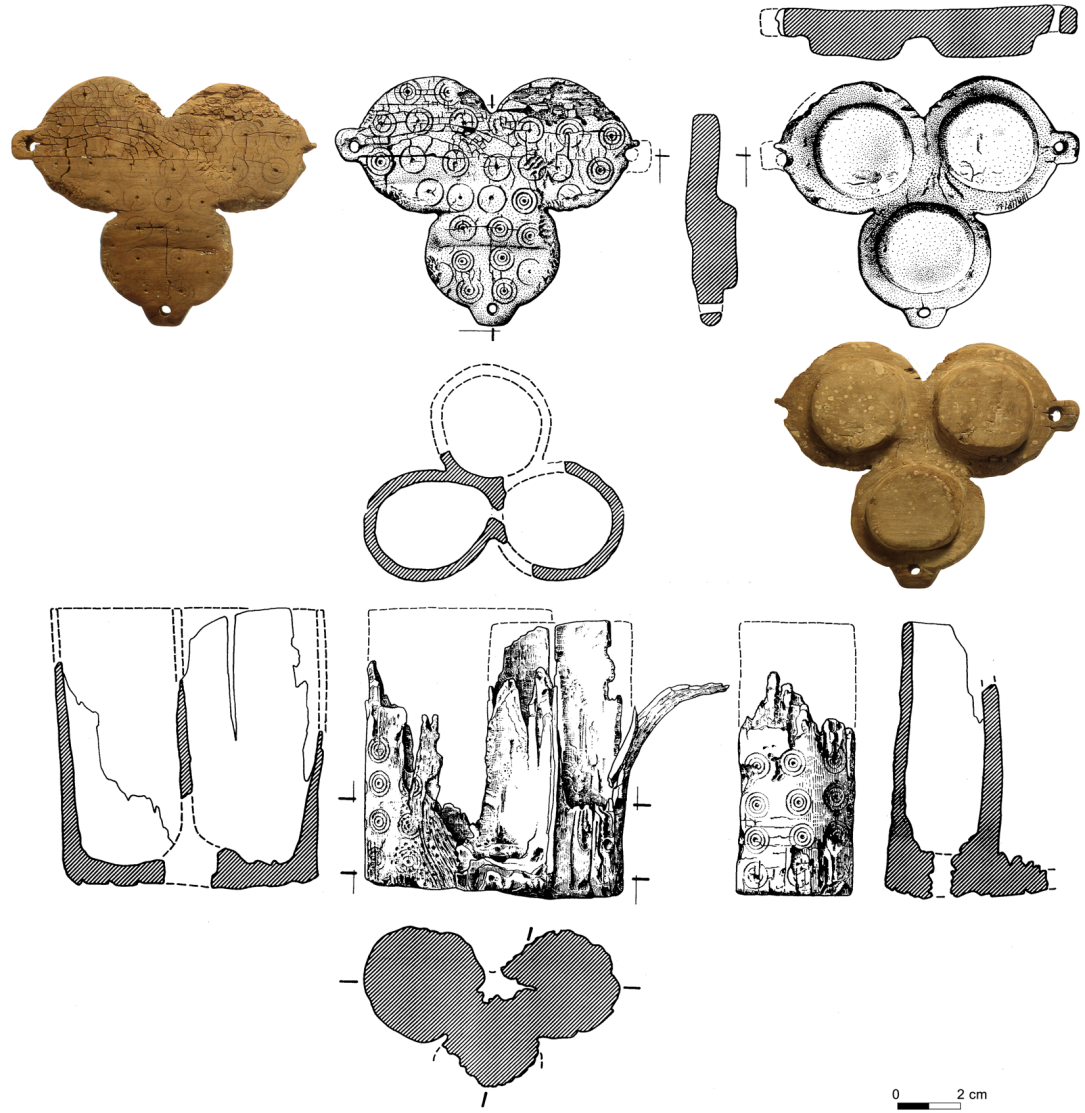
Trilobed container from Chamber 5 hosting the hair strands analysed (drawing by R. Álvarez; photo P. Witte).

The containers in Chamber 5 held locks of human hair which were up to 13 cm long and presented a reddish color^95^. The hair strands analyzed in this study come from one of the three compartments of the only container found which was made of olive tree wood (*Olea europaea*) (Fig. 3). This highly sophisticated piece of Prehistoric woodcraft was closed by means of a trilobed lid which had been carved out of boxwood (*Buxus* cf. *balearica*) and did not require an independent base. Both the lid and the outer walls of the container showed the typical pattern of one or more concentric circles surrounding a central dot. Wear traces abound and they are responsible for the partial erasure of some of the circles. Moreover, characteristic wear traces were found in two of the three perforated lugs of the lid, suggesting that the third had remained tied to the tube by means of a string each time the lid was opened. It thus seems likely that the container was opened and closed multiple times and, therefore, it is also possible that the hair found inside came from different mortuary events corresponding to different individuals. Two AMS radiocarbon dates for this container, on wood (OxA-5772: 2810 ± 65 BP) and human hair (OxA-8263: 2585 ± 40 BP) samples respectively, indicate its use in the early 1st millennium cal BCE, in accordance with two other absolute dates from the same deposit obtained from human hair samples (Methods).

### Detection of alkaloids in hair strands

**Figure 4:**
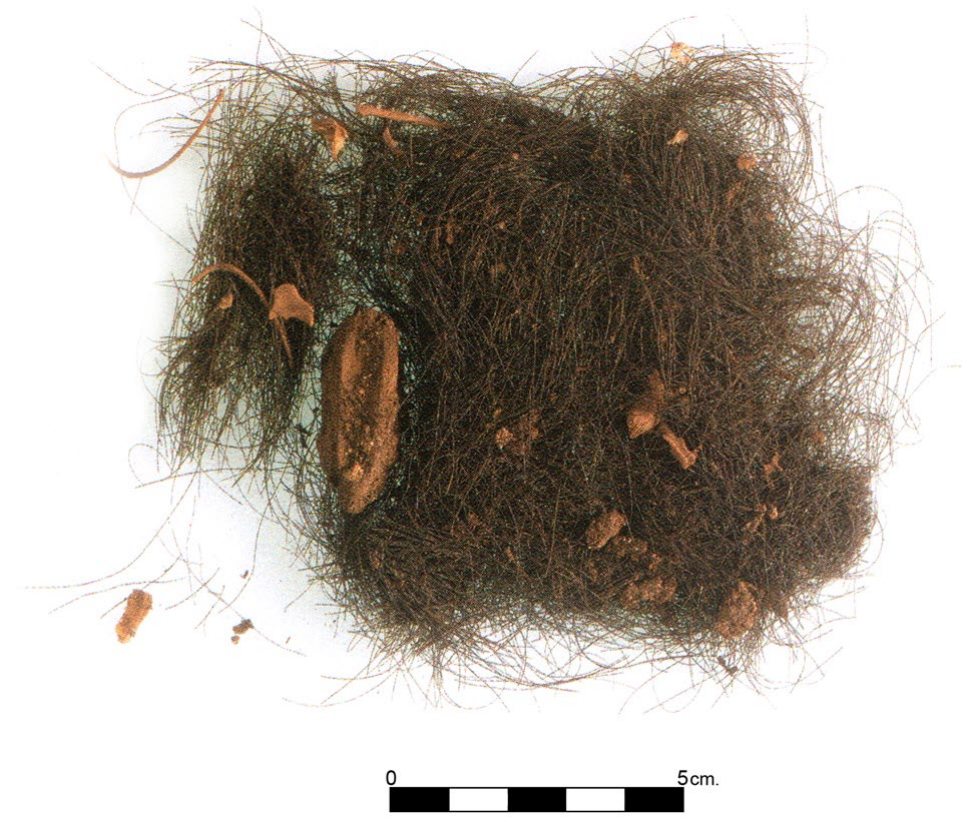
Human hair strands deposited in the trilobed container, and some microfaunal bones attached to the locks (Photo P. Witte).

Hair testing has revealed itself as an effective method to detect the consumption of certain drugs and is a widely-accepted technique in the field of Forensic Toxicology^96^. In recent years, chemical analysis of prehistoric human hair has been successfully applied to different cultural contexts^63–67,70–72,74^. The study of drug use in Prehistoric Europe has mainly been based on indirect evidence, such as archaeobotanical remains of drug plants, artistic depictions, and occasionally the detection of drug alkaloids in certain artefacts^97^. The unusual finding of human hair in the cave of Es Càrritx provided the opportunity to obtain direct evidence for the use of plant drugs by Late Bronze Age people. The hair strands analyzed were found inside a container from Chamber 5 (Fig. 4). The complete absence of hair bulbs, as expected from the ritual described above, prevented the determination of sex of the hair strands by means of aDNA^98^.

The flora native to Menorca includes the psychoactive species *Datura stramonium*, *Hyoscyamus albus* and *Mandragora automnalis* which contain the tropane derivatives atropine and scopolamine, *Ephedra fragilis* which contains the phenylethylamine derivative ephedrine, and *Papaver somniferum* which contains a variety of benzylisoquinoline alkaloids, morphine and papaverine among them^99,100^.

Some of these plant species have been found at various archaeological sites in Europe. Wild opium poppy (*Papaver somniferum* subsp. *setigerum*) is currently distributed throughout the central and western Mediterranean^101^, including Menorca. The domestication of opium poppy likely took place in the Western Mediterranean during the Early Neolithic: archaeobotanical remains of the cultivated variety (*Papaver somniferum* subsp. *somniferum* L.) have been recovered in several sites in Italy, southern France and Spain^102^. Then this species spread rapidly all over Europe, but no archaeobotanical remains of the plant have been found in the prehistoric record of the Balearics. Charred capsules of *Datura stramonium* were recovered in a Middle Bronze Age ritual pit at Prats, Andorra, *ca.* 1600 BCE^103^ but paleobotanical evidence is absent in prehistoric Menorca. However, a seed of *Hyoscyamus* sp. was recovered in the Hypogeum 3 of S’Alblegall, a rock-cut tomb dated to ca. 1450 cal BCE^104^. Archaeopalynological data from Es Forat de ses Aritges, a burial cave located only a few meters from the cave of Es Càrritx, revealed the presence of *Ephedra fragilis* in samples dated ca. 1050 cal BCE^105^. Pollen of *Ephedra* sp. was identified in some ceramic vessels recovered at the ceremonial and funerary staggered tower-like structure of Son Ferrer, in Mallorca^106^, but the typology of the ceramics cannot be dated earlier than the sixth century BCE^107^. Traces of ephedrine have also been reported in the seeds of a yew tree species (*Taxus* sp.), but yew has not been found in the vegetation of Menorca in the Bronze Age. While an amorphous fragment of carved wood from Hypogeum XXI of the Calescoves cemetery was made of *Taxus baccata,* it was proposed that the wood itself was imported to Menorca either as raw material or as a manufactured object^108^.

The present study on hair samples from the cave of Es Càrritx focused on the analysis of atropine, scopolamine, and ephedrine. The method of choice was Ultra-Performance Liquid Chromatography coupled to High Resolution Mass Spectrometry (UPLC-HRMS), a highly sensitive and selective technique for monitoring specific ions. The alkaloids were monitored through their molecular ions: *m/z* 290.175 (atropine), 304.155 (scopolamine), and 166.123 (ephedrine). Standards were obtained from Sigma-Aldrich Co., St Louis, MO, USA (atropine and scopolamine), and from Laboratorio Biosano S.A., Chile (ephedrine). Solvents were chromatographic grade from Merck (Darmstadt, Germany). Morphine was excluded from the study since it has been shown to be unstable in archaeological contexts^37,109^ and papaverine, another compound characteristic of poppy seeds, has shown carryover effects in liquid chromatography systems rendering normal analyses untrustworthy^37^.

## Results

**Figure 5:**
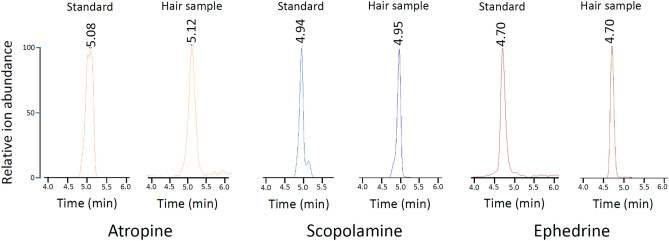
UHPLC-HRMS results from the analyses of alkaloid standards at 1000 pg/µL and of one of the three replicated hair samples from the cave of Es Càrritx (10.1 mg). Alkaloids were monitored at *m/z* = 290.175 (atropine), *m/z* = 304.155 (scopolamine) and *m/z* = 166.123 (ephedrine).

Linear calibration lines were obtained for pure alkaloids: Y = 8.20 × 10^5^ + 3.60 × 10^4^ * X, R^2^ = 0.999; Y = 2.15 × 10^5^ + 1.93 × 10^4^ * X, R^2^ = 0.990; and Y = 1.51 × 10^6^ + 1.64 × 10^4^ * X, R^2^ = 0.999 for atropine, scopolamine, and ephedrine, respectively, where Y is the instrument ion counts and X the concentration of the analyte in pg alkaloid/µL. The limits of detection were 0.1, < 0.1 and < 0.1 pg alkaloid/mg hair and the limit of quantitation were 1, < 1 and < 1 pg alkaloid/mg hair for atropine, scopolamine and ephedrine, respectively. The alkaloids in the hair extracts were identified by the appearance in the chromatogram of peaks at the same molecular masses and retention times as the alkaloid standards. The analysis showed the presence of atropine, scopolamine and ephedrine in the three replicated hair samples (Fig. 5) at the following concentrations: 6.7, 9.2, and 10.7 (mean = 8.9) pg atropine/mg hair, 384, 423 and 504 (mean = 437) pg scopolamine/mg hair, and 295, 328 and 367 (mean = 330) pg ephedrine/mg hair.

## Discussion

The most common theory of drug incorporation into the hair matrix is that it takes place at the root level. As chemicals circulate in the blood stream, they are incorporated in the growing hair matrix at the base of the follicle^110^. Therefore, hair analysis can provide a historical profile of an individual’s exposure to the substances, over a period of weeks to months depending on the length of hair collected^111^. Modern human scalp hair grows at an average rate of 1 cm/month depending on hair type, phenotypic affiliation, sex and age^112^. The length of the hair strands and the analysis of segments all along the hair shafts point to consumption over a period of nearly a year; hence, drug intake was sustained over time probably well before death. A more exact timing of consumption may be inferred from segmental hair analysis. However, such analysis could not be performed since the lack of bulbs in the hair strands prevented a clear distinction between distal and apical segments.

The three alkaloids monitored were found in the cave of Es Càrritx hair analyzed, ephedrine and scopolamine at higher concentrations than atropine. To our knowledge, no quantitative data on the consumption of these alkaloids by past populations has been reported; hence, the present data were compared with the few published reports on modern consumers.

Recent analysis of hair of patients suffering from *Datura* poisoning revealed atropine (8.4–15.0 pg/mg) and scopolamine (1.0–1.3 pg/mg) in a patient who admitted regular consumption of *Datura stramonium*^113^, and only scopolamine (14 to 48 pg/mg) in a regular *Cannabis* abuser who had consumed six dried flowers of *D. inoxia* in hot water^114^. These data are consistent with phytochemical studies that show that the ratio of scopolamine to atropine is substantially higher in *D. inoxia* than in *D. stramonium*^115,116^. The high ratio of scopolamine to atropine found in the hair from the cave of Es Càrritx cannot be associated with the use of *D. inoxia* since this is not a species native to the Old World. A likely candidate is *D. stramonium* since chemical analyses of different tissues from different varieties of this species at different ontogenetic stages have shown that the ratio of scopolamine to atropine varies substantially from less than one in stems of senescent plants^117,118^ to values as high as 12 in leaves of young plants^119^.

Ephedrine was detected in the hair of two bodybuilders (670 and 10,700 pg/mg) who used it as doping agent^120^, and in the hair of volunteers (2,254 pg/mg) who had been given oral doses of the alkaloid^121^. A phytochemical study of the genus *Ephedra* showed that, of all species studied, *E. fragilis* was the one with highest concentration of ephedrine^122^, and this is only *Ephedra* native species growing in Menorca^99,100^. The concentration of ephedrine in the hair from the cave of Es Càrritx (mean 330 pg/mg) was lower than that found in modern consumers by nearly an order of magnitude. Ephedrine was consumed as plant material in the past and pure compounds in contemporary times. The referred volunteers were given three daily doses of 50 mg ephedrine^121^. Since the concentration of ephedrine in *E. fragilis* is 21 mg/g dry plant tissue^122^, close to one gram of dry plant tissue, either in one or in multiple events during the period of accumulation in the cave of Es Càrritx hair strands, would have been consumed to achieve such levels of ephedrine. Given that mean water content of Mediterranean woody species varies between 30 and 70%^123^, this would imply that ephedrine in the hair from the cave of Es Càrritx would arise from some 2 g of wet plant material, a reasonable amount to collect and process.

### Pharmacology and possible uses of the alkaloids found

Tropane alkaloids are highly psychoactive, exerting multiple effects on the central nervous system. Rather than just being hallucinogens, atropine and scopolamine belong to the group of deliriant drugs, i.e., they induce delirium characterized by extreme mental confusion, strong and realistic hallucinations, disorientation, alteration of sensorial perception, and behavioral disorganization^124^. Out-of-body experiences and a feeling of alteration of the skin, as if growing fur or feathers, are usually reported^125^. In Europe, tropane containing Solanaceae have a long history of use as medicines, poisons, and intoxicants, but they have achieved their most notorious reputation in association with European witchcraft during the Middle Ages/Early Modern period. Allegedly, witches smeared themselves with certain unguents to help them fly to demonic Sabbaths (hence their name of flying ointments) or be transformed into animals, according to some testimonies. However, it seems clear that people had hallucinatory experiences using such ointments, the main ingredients of which likely being tropane-containing Solanaceae^126–129^. The powerful mental and behavioral effects of these plants have made them indispensable ingredients in the botanical preparations used by shamans worldwide in rituals for divination, prophecy, and ecstasy^124^.

On the other hand, ephedrine exerts a sympathomimetic action similar to that of adrenaline, i.e., excitement and enhancement of mental alertness and physical activity, reduction of fatigue, improvement of concentration, and suppression of hunger. It has also served as a remedy to treat colds, asthma, and hay fever among other medical purposes^130^.

The use of plants in the past may be deduced from the archaeological context or ethnobotanical data^131^. Several Bronze Age burial sites and ceremonial places in the Balearic Islands revealed the deposition of flowers and the use of aromatic species to create a sensory experience^93,94,106,132,133^. However, psychoactive plants were not found in such sites and places nor in any domestic context. Only the cave of S’Alblegall has provided an isolated seed of *Hyoscyamus* sp., but the occurrence of this wild plant has been interpreted as unintentional by the excavators^104^, probably being a weed from crop processing which was accidentally harvested^134^. Neither at the cave of Es Càrritx, systematically sampled for botanical material, is there archaeobotanical evidence of the plant species containing the alkaloids detected in the hair samples^93,94^. Thus, the use of drug plants was excluded from the burial rites.

The Bronze Age populations of Menorca may have employed drug plants for their medicinal properties. In the Old World, the use of mandrake as a sedative and to induce pain relief for surgical procedures can be traced back for over two millennia^135^. It is interesting to note the occurrence at the cave of Es Càrritx of three trepanned skulls, belonging to adult males and all of them with clear signs of survival even for over a year^90^, although the relation between the sampled hair strands and the trepanned skulls cannot be safely established. At any rate, it is likely that plants involved in medicinal practices were used outside the funerary environment, and hence their residues are not to be found at funerary sites. According to ethnobotanical sources from Menorca, *Ephedra* leaves are currently employed in several treatments, cigarettes prepared with *Datura stramonium* and *Hyoscyamus albus* have been smoked as an anti-asthmatic, and *Mandragora* roots were added to balms to cure insomnia^136–138^.

Given the disparity between the number of individuals and that of hair-carrying containers found in the cave of Es Càrritx, and the fact that no political or economic privileges have been found among Late Bronze Age people from Menorca^139^, the tonsure ritual should be explained by other factors. At the end of the second millennium BCE (*ca*. 1200–1000 cal BCE) there is evidence of the celebration of shamanic ceremonies inside the nearby cave of Es Mussol. A collection of wooden objects was discovered in a small chamber, which included two carvings made of *Olea europea* wood, one depicting a man’s head, face, and neck, while the other is a zoo-anthropomorphic figurine presenting two incipient stag antlers in the upper part of its head (a Prehistoric precedent of the later Celtic god Cernunnos?)^89,140^. Could this transformation into an animal be related to the employment of psychotropic alkaloid containing plants during ritual ceremonies? If so, then, their officiators might have performed these ceremonies under the effects of hallucinogenic plants. And perhaps, these distinct individuals received a special funerary treatment in acknowledgment of their shamanic character.

## Concluding remarks

As early as the Paleolithic period, humans came across the non-food properties of certain plants. The results presented here indicate that several alkaloid-bearing plants were consumed by Bronze Age people from Menorca (although Solanaceae and *Ephedra* were not the only ones to have been consumed). Interestingly, the psychoactive substances detected in this study are not suitable for alleviating the pain involved in severe palaeopathological conditions attested in the population buried in the cave of Es Càrritx, such as periapical abscesses, severe caries and arthropathies^90^. Considering the potential toxicity of the alkaloids found in the hair, their handling, use, and applications represented highly specialized knowledge. This knowledge was typically possessed by shamans^141^, who were capable of controlling the side-effects of the plant drugs through an ecstasy that made diagnosis or divination possible^124^.

An interesting parallelism may be drawn with the series of concentric circles carved on the lids of the tubes found in the cave of Es Càrritx. Given the mydriatic effect of the alkaloids detected in the hair samples, these circles may be interpreted as eyes , which somescholars have considered as a metaphor of inner vision, in some cases related to altered states of consciousness and visionary experiences under the influence of hallucinogens^142–144^. The recent discovery that two Late Pre-Columbian ceramic containers from the Central Arkansas River Valley which tested positive for atropine, were decorated with spiral motifs^145^ supports this interpretation.

By *ca*. 800 cal BCE, populations at the Balearic Islands underwent a transformation of its social structures. Archaeological evidence points to demographic growth, abandonment of the burial places, and a slight decrease in extra-insular contacts. In this context, in the cave of Es Càrritx, some individuals reluctant to abandon ancient traditions, concealed a collection of ritual objects belonging to certain members of the community, possibly shamans, in the hope that the former social order could be re-established in the future. And the best location to assure the protection of the assemblage was found going deeper inside the burial ground of the ancestors.

## Methods

### Radiocarbon dating

**Figure 6:**
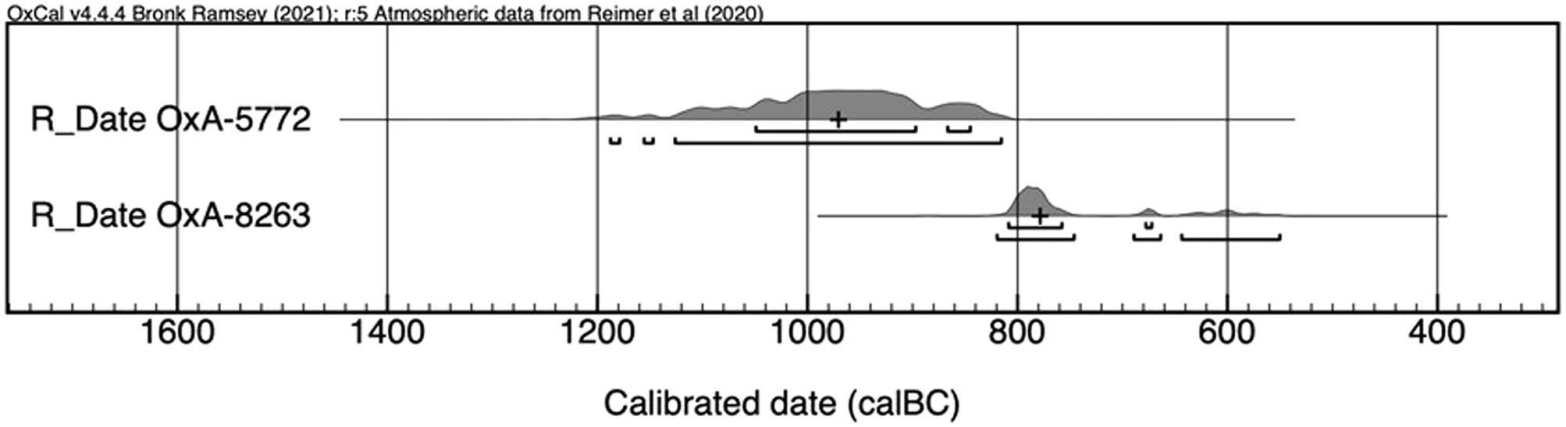
Calibration of the two samples for the trilobed container with OxCal v.4.4.4, and IntCal20 atmospheric curve.

Two radiocarbon dates on human hair samples from the hoard found in Chamber 5 but from different tubes were previously published: OxA-7235, 2935 ± 45 BP^146^ and Beta-125220, 2820 ± 50 BP^89^. Both are in accordance with Late Bronze Age chronology. The radiocarbon dates directly related to the research presented here correspond to the human hair sample analysed , and to a wood sample from the trilobed lid (Table 1). The radiocarbon result of the human hair sample (OxA-8263, 2585 ± 40 BP) shows a high probability range in the late 9th and early 8th century cal BCE. It is worth noting that OxA-8263 was obtained after redating the same hair sample that had produced the date OxA-5773, 2445 ± 50 BP^146,147^, as this result was considered too modern for its archaeological context. A small fragment of wood detached from the trilobed lid containing the hair analysed here (OxA-5772, 2810 ± 65 BP)^147^ dates to the beginning of the 1st millennium cal BCE. Its earlier temporality in comparison with OxA-8263 could be explained considering that wood is a long-life sample and that the container was produced and used before it was finally filled with the hair analysed and subsequently hoarded under the soil of Chamber 5 (Table 1; Fig. 6).

**Table 1:** Valid radiocarbon dates for the trilobed lid from Chamber 5 of the cave of Es Càrritx^89,146,147^ (calibration using OxCal v4.4.4 and applying IntCal20 calibration curve)^148,149^.

Sampled material	14C id	BP	cal BCE 1*s*	cal BCE 2*s*	cal BCE (median)
Wood	OxA-5772	2810 ± 65	1050–846	1188–816	971
Human hair	OxA-8263	2585 ± 40	809–673	820–551	779

### Preparation of extracts and chemical analysis

The analysis of the hair strands was carried out in accordance with relevant guidelines and regulations for prehistoric human tissue.

Nitrile gloves were used during manipulation of hair in the laboratory and all equipment and glassware were washed with neutral pH detergent, distilled water and alcohol before use. A lock of hair strands was minced with the use of scissors, resulting in hair sections shorter than 1 mm. Each of three replicated hair samples (9.9, 10.1 and 10.2 mg) was washed with dichloromethane (3 × 3 mL for 10 min) and dried with a nitrogen flow. After addition of alkali to the dry extract (400 µL of aqueous 2.5 M NaOH), the suspension was sonicated for 2 h at 50° and thereafter extracted with dichloromethane (3 × 400 μL). The combined organic phases were mixed with acid (500 μL of 25 mM HCl in methanol), dried with a nitrogen flow, dissolved in methanol, and transferred to an insert inside the sample vial (100 µL final volume).

The UHPLC-HRMS system consisted of an LPG-3400RS quaternary pump, a WPS-3000TRS autosampler, a TCC-3000RS column oven, a high resolution Orbitrap Exactive Plus mass spectrometric detector equipped with electrospray ionization (ESI), and Xcalibur 3.1 software, all from Thermo Scientific, Bremen, Germany. An Acquity UHPLC HSS T3 (1.8 μm particle size, 2.1 mm internal diameter × 100 mm length, Waters, Milford, MA, USA) analytical column was used. Auto sampler temperature was 4 °C, column temperature 35 °C, capillary temperature 300 °C, auxiliary gas heater temperature 200 °C, injector temperature 15 °C, injection volume 10 µL. The detector was used in the positive ion mode scanning between *m/z* 80 and 1000 with resolution 140,000 and the following conditions: S-lens RF level 50, spray voltage 6 kV, sheath gas flow rate 35 (arbitrary units), auxiliary gas flow rate 5 (arbitrary units), sweep gas flow rate 1 (arbitrary units). Solvents were analytical chromatographic grade (Merck, Darmstadt, Germany). The mobile phases were: A) 20 mM ammonium acetate + 0.1% formic acid in aqueous solution, and B) 20 mM ammonium acetate + 0.1% formic acid in acetonitrile solution. The linear gradient program [time (min), %A] was: [0, 98], [4, 98], [6, 5], [7, 98] and [10, 98].

Approximate concentrations of alkaloids in hair were estimated from calibration lines obtained with neat standards at 0.1, 1, 10, 100, and 1000 pg/µL. The limit of detection for each alkaloid was defined as the minimum concentration of standard solution where signal-to-noise (S/N) ratio was less than 3 in the interval ± 2 min around the alkaloid peak. Similarly, the limit of quantitation for each alkaloid was defined as the minimum concentration of standard solution where S/N was less than 10.

## Data Availability

All data generated during this study are included in this manuscript.

## Code availability

All software used for data analysis is indicated in the Methods section.
